# What is the optimal number of embryos to transfer for POSEIDON group 1 and group 2? A retrospective study

**DOI:** 10.1186/s13048-024-01443-y

**Published:** 2024-05-31

**Authors:** Rang Liu, Qiuju Zhang, Lan Geng, Huiqing He, Chang Xu, Jiali Feng, Miaoling Song, Yanpei Cao, Tianren Wang, Xi Xia

**Affiliations:** 1 Center for Reproductive Medicine, Peking University Shenzhen Hospital, No. 1120 Lotus Road, Futian District, Shenzhen, Guangdong 518036 China; 2 Shantou University Medical College, Shantou, Guangdong 515000 China; 3 Intelligence Hospital Research Academy, Peking University Shenzhen Hospital, Shenzhen, Guangdong 518036 China; 4 Center for Reproductive Medicine, The University of Hong Kong-Shenzhen Hospital, Shenzhen, Guangdong 518000 China

**Keywords:** Embryo transfer strategy, POSEIDON criteria, Low prognosis, Live birth rate, Multiple birth rate

## Abstract

**Background:**

The 2016 Patient-Oriented Strategy Encompassing IndividualizeD Oocyte Number (POSEIDON) criteria redefined the poor responders as low prognosis patients. The embryo transfer strategy for POSEIDON patients remained to be addressed. This study aimed to investigate the optimized number of embryos to transfer for unexpected low-prognosis patients (POSEIDON Group 1 and Group 2) with blastocyst transfer in their first frozen cycle.

**Methods:**

A retrospective cohort study of 2970 patients who underwent frozen-thawed embryo transfer (FET) between January 2018 and December 2021. Patients from POSEIDON Group 1 (*N* = 219) and Group 2 (*N* = 135) who underwent blastocyst transfer in their first FET cycles were included and divided into the elective single embryo transfer (eSET) group and the double embryo transfer (DET) group.

**Results:**

For POSEIDON Group 1, the live birth rate per embryo transfer of the DET group was slightly higher than the eSET group (52.17% vs 46.15%, OR 0.786, 95% CI 0.462–1.337, *P* = 0.374; adjusted OR (aOR) 0.622, 95% CI 0.340–1.140, *P* = 0.124), while a significant increase of 20.00% in the multiple birth rate was shown. For Group 2, higher live birth rates were observed in the DET group compared to the eSET group (38.46% vs 20.48%, OR 0.412, 95% CI 0.190–0.892, *P* = 0.024; aOR 0.358, 95% CI 0.155–0.828, *P* = 0.016). The difference in the multiple birth rate was 20.00% without statistical significance. Univariate and multivariate analyses revealed that age (OR 0.759, 95% CI .624–0.922, *P* = 0.006 and OR 0.751, 95% CI 0.605–0.932, *P* = 0.009) and the number of transferred embryos (OR 0.412, 95% CI 0.190–0.892, *P* = 0.024 and OR 0.367, 95% CI 0.161–0.840, *P* = 0.018) were significant variables for the live birth rate in POSEIDON Group 2.

**Conclusions:**

The findings in the present study showed that eSET was preferred in the first frozen cycle for POSEIDON Group 1 to avoid unnecessary risks. Double embryo transfer strategy could be considered to improve the success rate for POSEIDON Group 2 with caution. Further stratification by age is needed for a more scientific discussion about the embryo transfer strategy for POSEIDON patients.

**Supplementary Information:**

The online version contains supplementary material available at 10.1186/s13048-024-01443-y.

## Background

Despite the spectacular development and fruitful achievement, assisted reproductive technology (ART) has not yet perfectly satisfied every patient. Patients with diminished ovarian reserve (DOR) or poor ovarian response (POR) are considered one of the most difficult populations for their frustrating outcomes even after ART treatment, making their management a thorny issue for reproductive clinicians [1]. To address the heterogeneity in the definition, in 2011 the European Society of Human Reproduction and Embryology (ESHRE) standardized poor responders by the Bologna criteria [2]. It was pointed out that the Bologna criteria might not be practical or efficient enough to manage patients since heterogeneity of treatment strategy remained after categorization. The 2016 Patient-Oriented Strategy Encompassing IndividualizeD Oocyte Number (POSEIDON) group more detailly stratified the poor responders into 4 groups by age, ovarian biomarkers, and the number of oocytes retrieved, transiting the definition of POR to the concept of low prognosis. Under the new criteria, patients are further classified as unexpected low-prognosis if they presented a favorable ovarian reserve test (antral follicle count (AFC) ≥ 5 or/and anti-mullerian hormone (AMH) ≥ 1.2 ng/ml) but with a low response in a standard ovarian stimulation cycle (≤ 9 oocytes), and as expected low-prognosis if low ovarian reserve were shown [3].

Although POSEIDON stratification ameliorates the difficulties in counseling and prognosis prediction, it continued to challenge experts that how to manage these low-prognosis patients in each node of ART treatment to optimize the outcomes. Scholars have intensely discussed the pretreatment, adjuvant treatment, ovarian stimulation protocols, and maximization of oocyte or embryo usability by laboratory techniques [1]. Up to the present, research has yet to pay much attention to the embryo transfer strategy for low-prognosis patients under the POSEIDON classification, which is a question eventually inevitable to be answered.

As assisted conception technologies progressed, high-quality success was more and more pursued rather than just obtaining live births. Ideally, successful in vitro fertilization (IVF) treatment was expected to result in one healthy singleton by an optimal embryo transfer strategy. However, in order to improve the prognosis of unfavorable patients, the transfer of more than one embryo would be considered, which was also out of the wishes for reducing unnecessary multiple transfers and relieving the patient’s anxiety. Unfortunately, patients were exposed to the potential risk of multiple births. While maintaining a satisfactory delivery rate, multiple births should be controlled by various strategies to reduce complications, including adverse maternal and neonatal outcomes [4]. Early in the end of twentieth century, elective single embryo transfer (eSET) was recommended to mitigate the risk of multiple births [5]. The 2013 American Society for Reproductive Medicine and Society for Assisted Reproductive Technology (ASRM/SART) committee opinion suggested young patients with favorable prognoses be transferred 1–2 cleavage-stage embryos or single blastocyst [6]. Further defining the concept of “favorable” in detail, the 2021 ASRM/SART committee opinion recommended single embryo transfer for patients ≤ 37 years with favorable prognoses, and that increased embryo number by age or if the embryo was not euploid or favorable [7]. Since 42%-45% of favorable prognosis patients in the US did not undergo eSET, leading to an unacceptable multiple birth rate, it was recently proposed to enhance adherence to the guideline [8, 9].

Paradoxical as it may seem, patients classified as low prognosis by POSEIDON criteria undergoing blastocyst transfer in their first frozen embryo transfer cycle (FET) were associated with a favorable prognosis according to the 2021 ASRM/SART committee opinion [7]. The appropriate number of embryos to transfer for this patient population remains to be addressed. Thus, the aim of this article was to investigate the optimized number of transferred embryos to improve the prognosis of unexpected low-prognosis patients by comparing the clinical outcomes of different blastocyst transfer protocols in the first FET among POSEIDON Groups 1 and 2.

## Materials and methods

### Patient selection

**Fig. 1:**
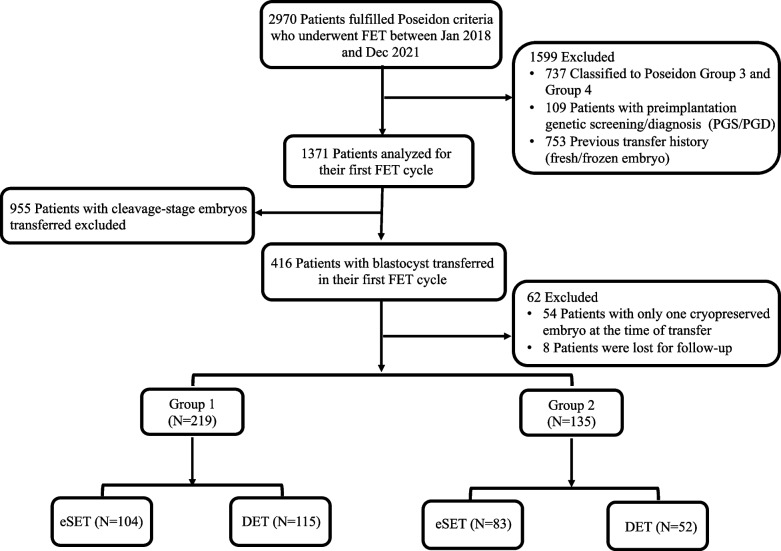
The flowchart of the study design

This was a retrospective study conducted among patients who underwent ART treatment at the Peking University Shenzhen Hospital Reproductive Medicine Center between January 2018 and December 2021. 2970 patients fulfilling the POSEIDON criteria who underwent FET cycles were sorted out and classified accordingly [3]. Group 1: Patients <35 years with AFC ≥5 and/or AMH ≥1.2 ng/mL and with an unexpected poor or suboptimal ovarian response (retrieved ≤9 oocytes). Group 2: Patients ≥35 years with AFC ≥5 and/or AMH ≥1.2 ng/mL and with an unexpected poor or suboptimal ovarian response. As reported in a previous study, AMH and AFC had equivalent accuracy and efficacy for patient stratification [10]. In our study, AMH rather than AFC was used for grouping since it was more objective with less potential bias. Oocyte retrieval in Group 1 or Group 2 was conducted under standard ovarian stimulation. Patients classified to POSEIDON Group 3 and Group 4, who underwent preimplantation genetic screening/diagnosis (PGS/PGD), who were with previous fresh embryo transfers or FET history were excluded. Patients with only one cryopreserved embryo at the time of transfer were also eliminated. Only patients from Group 1 and Group 2 with blastocysts transferred in their first FET cycle and had completed the follow-up were included for analysis. Outcome follow-ups were conducted offline when patients returned to the center or online by telephone. Based on the number of blastocysts to transfer, patients were further divided into subgroups including elective single embryo transfer (e-SET) and double embryo transfer (DET) (Fig. 1).

### Ovarian stimulation and embryo transfer

Standard ovarian stimulation protocols including gonadotrophin-releasing hormone (GnRH) antagonist and GnRH agonist (ultra-long/long) were used individually [11–13]. Based on ovarian reserve, age, body mass index (BMI), and previous stimulation outcome if available, patients received subcutaneous injections of recombinant gonadotropin (recombinant FSH (GONAL-f, Merck Serono, Germany) /purified hMG (Urofollitropin, Livzon Pharmaceuticals, China) /recombinant FSH plus hMG or recombinant LH (Luveris, Merck Serono, Germany), 2:1 ratio) with an initial daily dosage of 150-450IU. GnRH-antagonist (Cetrotide, Merck Serono, Germany or Ganirelix, MSD, USA) was administered starting from the day when the dominant follicle reached 12-14 mm in diameter until the trigger day. GnRH-agonist (Decapeptyl, Ferring pharmaceuticals, Switzerland) was initiated prior to ovarian stimulation with one to three injections to achieve downregulation in the ultra-long protocol or from the previous mid-luteal phase in the long protocol. Recombinant HCG (Choriogonadotropin alfa, Merck Serono, Germany) or recombinant HCG combined with GnRH agonist was applied to trigger the final maturation when at least two lead follicles reached greater than 18 mm in diameter. Oocyte retrieval was conducted 36-38 hours after triggering. Blastocyst assessment was conducted on the day of freezing and before transfer using the Gardner scoring system. Embryo transfer decisions were made based on the clinical situation with informed consent. Considerations included age, quantity and quality of the available embryos, and personal willingness.

### Outcomes

The primary outcomes were the live birth rate, defined as the rate of patients giving birth to a fetus of any living sign after 28 weeks of gestation, and the multiple birth rate, defined as the rate of giving birth to more than one living infant among all live births. The secondary outcomes included (i) biochemical pregnancy rate defined as the rate of patients with positive detection of urine or serum beta-HCG test, (ii) clinical pregnancy rate, defined as the rate of patients with pregnancy diagnosed by ultrasonographic confirmation of intrauterine gestational sac 28 days after embryo transfer, (iii) ongoing pregnancy rate, defined as the rate of patient with ultrasonographic confirmation of intrauterine fetal heart activity beyond 12 weeks of gestation.

### Statistics

All the statistical analysis was performed by SPSS statistics version 26.0 (IBM, Armonk, NY, USA). Continuous data were expressed as mean ± SD and compared by Student’s t-test. Categorical data were presented as frequencies and percentages. The Chi-square test or Fisher’s exact test was used to compare rates in the baseline characteristics. Logistic regression analysis was applied to output the unadjusted odds ratio (OR) and the adjusted OR (aOR) with 95% CI for the rates of outcomes. Potential confounding variables were adjusted including age, BMI, indications for IVF, AFC, endometrium thickness (EMT) on the day of transfer, and the number of oocytes retrieved. Finally, univariate and multivariate analyses were performed to analyze the associated variables for live birth rates and the results were reported as OR with 95% CI. A two-sided *P* value <0.05 was considered statistically significant.

## Results

### Baseline clinical characteristics

In this study, a total of 354 frozen blastocyst transfer cycles from POSEIDON Group 1 (*N* = 219) and Group 2 (*N* = 135) were analyzed. Supplementary Table 1 summarized general patient characteristics. Compared to POSEIDON Group 1, Group 2 had significantly higher age (37.87±2.52 versus 30.68±2.70, *P* = 0.000), lower AFC (9.95±4.65 versus 12.01±5.13, *P* = 0.000), lower AMH (3.00±2.32 versus 3.77±2.25, *P* = 0.002) and higher total gonadotropin (Gn) dose (2980.56±1161.65 versus 2454.37±1028.87, *P* = 0.000). Basal hormonal level (except LH), controlled ovarian hyperstimulation (COH) protocol, and the number of oocytes retrieved were comparable between Group 1 and Group 2. Indications for IVF (*P* = 0.028) and BMI (22.14±2.53 versus 21.18±3.02, *P* = 0.002) were significantly different between groups.

The number of patients/cycles transferring one embryo and transferring two embryos was 104 versus 115 in POSEIDON Group 1 and 83 versus 52 in Group 2, respectively. The baseline characteristics of subgroups were summarized in Table 1. Significant differences existed in comparison of indications for IVF (*P* = 0.001), AFC (11.20±4.95 versus 12.74±5.21, *P* = 0.027), and number of oocytes retrieved (6.25±2.18 versus 7.03±1.89, *P* = 0.005), EMT on the day of transfer (11.54±2.26 versus 10.89±2.28, *P* = 0.037) between eSET and DET in Group 1. All indicators were comparable between eSET and DET within Group 2.

**Table 1:** Baseline clinical characteristics of patients subgrouped by transferred embryo number in the first FET cycle

	Group 1 (*n* = 219)			Group 2 (*n* = 135)		
	eSET (*n* = 104)	DET (*n* = 115)	*P* value	eSET (*n* = 83)	DET (*n* = 52)	*P* value
**Age (years)**	30.35 ± 2.71	30.98 ± 2.67	0.082	37.82 ± 2.60	37.96 ± 2.41	0.751
**BMI (kg/m2)**	20.88 ± 3.08	21.45 ± 2.95	0.165	22.44 ± 2.59	21.67 ± 2.38	0.084
**Duration of infertility(years)**	3.32 ± 1.60	3.41 ± 2.29	0.729	3.84 ± 3.05	4.08 ± 3.37	0.673
**Indications for IVF**						
** Tubal factor**	32.69%	45.22%	0.001	45.78%	48.08%	0.743
** Endometriosis**	4.81%	12.17%		1.20%	1.90%	
** Male factor**	7.69%	13.04%		13.25%	19.23%	
** Combined factors**	6.73%	7.83%		4.82%	5.77%	
** Unknown factors**	48.08%	21.74%		34.94%	25.00%	
**AFC**	11.20 ± 4.95	12.74 ± 5.21	0.027	10.18 ± 4.78	9.58 ± 4.44	0.464
**FSH (IU/L)**	7.82 ± 1.90	7.85 ± 2.41	0.932	7.82 ± 2.18	8.45 ± 2.80	0.146
**LH (IU/L)**	4.63 ± 2.66	5.10 ± 2.87	0.204	4.23 ± 2.03	4.19 ± 1.55	0.911
**E2 (pg/mL)**	49.89 ± 58.03	48.76 ± 30.41	0.855	45.38 ± 23.79	53.75 ± 34.65	0.098
**P (ng/mL)**	0.67 ± 0.91	0.83 ± 1.46	0.349	0.71 ± 1.08	0.83 ± 1.55	0.584
**PRL (ng/mL)**	22.78 ± 54.34	19.84 ± 26.58	0.607	22.02 ± 34.89	19.48 ± 27.97	0.658
**T (ng/mL)**	1.03 ± 5.52	0.93 ± 4.61	0.889	1.01 ± 3.82	0.54 ± 0.73	0.374
**AMH (ng/mL)**	3.48 ± 2.07	4.03 ± 2.38	0.069	3.08 ± 2.63	2.87 ± 1.74	0.610
**COH protocol**						
** GnRH-Agonist**	24.04%	31.30%	0.231	32.53%	23.08%	0.238
** GnRH-Antagonist**	75.96%	68.70%		67.47%	76.92%	
**Total Gn dose (IU)**	2549.06 ± 1026.15	2368.73 ± 1028.26	0.196	2934.10 ± 1155.65	3054.72 ± 1178.62	0.559
**No. Oocytes retrieved**	6.25 ± 2.18	7.03 ± 1.89	0.005	6.34 ± 2.04	6.60 ± 2.21	0.489
**EMT on the day of transfer (mm)**	11.54 ± 2.26	10.89 ± 2.28	0.037	10.67 ± 2.30	10.93 ± 1.95	0.509

*BMI* Body Mass Index, *IVF* In Vitro fertilization, *AFC* Antral Follicle Count, *FSH* Follicle-Stimulating Hormone, *LH* Luteinizing Hormone, *E2* Estradiol, *P* Progesterone, *PRL* Prolactin, *T* Testosterone, *AMH* Anti-Mullerian Hormone, *COH* Controlled Ovarian Hyperstimulation, *Gn* Gonadotropin

### Clinical outcomes

**Fig. 2:**
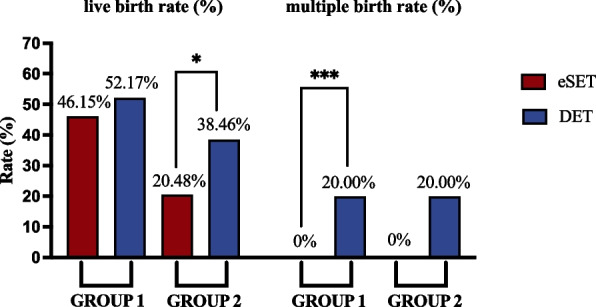
The primary outcomes of POSEIDON Group 1 and 2 with different transferred embryo numbers. eSET = elective single embryo transfer; DET = double embryo transfer

For POSEIDON Group 1, the live birth rate per embryo transfer was 46.15% in the eSET group and 52.17% in the DET group (OR 0.786, 95% CI 0.462-1.337, *P* = 0.374; aOR 0.622, 95% CI 0.340-1.140, *P* = 0.124). However, a multiple birth rate of 20.00% was shown in the DET group (*P* = 0.001) (Table 2, Fig. 2). Similarly, biochemical pregnancy rate, clinical pregnancy rate and ongoing pregnancy rate increased from 62.50% to 64.35% (OR 0. 923, 95% CI 0.532-1.602, *P* = 0.777; aOR 0.663, 95% CI 0.354-1.241, *P* = 0.199), 56.73% to 61.74% (OR 0.813, 95% CI 0.473-1.395, *P* = 0.451; aOR 0.585, 95% CI 0.315-1.089, *P* = 0.091), and 48.08% to 55.65% (OR 0.738, 95% CI 0.433-1.256, *P* = 0.263; aOR 0.614, 95% CI 0.335-1.123, *P* = 0.113) without statistically significant differences when comparing the eSET group to the DET group (Table 2).

**Table 2:** Pregnancy outcomes of first FET cycle (eSET versus DET)

	Group 1						Group 2					
	eSET (*N* = 104)	DET (*N* = 115)	Unadjusted group difference		Adjusted group difference^a^		eSET (*N* = 83)	DET (*N* = 52)	Unadjusted group difference		Adjusted group difference^a^	
			OR (95% CI)	*P* value	OR (95% CI)	*P* value			OR (95% CI)	*P* value	OR (95% CI)	*P* value
**Biochemical pregnancy rate (%)**	65 (62.50%)	74 (64.35%)	0.923 (0.532–1.602)	0.777	0.663 (0.354–1.241)	0.199	30 (36.14%)	29 (55.77%)	0.449 (0.221–0.910)	0.026	0.409 (0.189–0.888)	0.024
**Clinical pregnancy rate (%)**	59 (56.73%)	71 (61.74%)	0.813 (0.473–1.395)	0.451	0.585 (0.315–1.089)	0.091	28 (33.73%)	24 (50.00%)	0.509 (0.251–1.034)	0.062	0.473 (0.219–1.019)	0.056
**Ongoing pregnancy rate (%)**	50 (48.08%)	64 (55.65%)	0.738 (0.433–1.256)	0.263	0.614 (0.335–1.123)	0.113	17 (20.48%)	20 (38.46%)	0.412 (0.190–0.892)	0.024	0.358 (0.155–0.828)	0.016
**Live birth rate (%)**	48 (46.15%)	60 (52.17%)	0.786 (0.462–1.337)	0.374	0.622 (0.340–1.140)	0.124	17 (20.48%)	20 (38.46%)	0.412 (0.190–0.892)	0.024	0.358 (0.155–0.828)	0.016
**Multiple birth rate (%)**	0	12/60 (20.00%)	/	0.001	/	/	0	4/20 (20.00%)	/	0.155	/	/

^a^Adjusted for age, BMI, indications for IVF, AFC, EMT, No. oocytes retrieved

The live birth rate in the eSET group was significantly different from that in the DET group (20.48% versus 38.46%, OR 0.412, 95% CI 0.190–0.892, *P* = 0.024; aOR 0.358, 95% CI 0.155–0.828, *P* = 0.016) in POSEIDON Group 2. The multiple birth rate increased to 20.00% in the DET group without significance compared to the eSET group (*P* = 0.155) (Table 2, Fig. 2). The value for the ongoing pregnancy rate was in accord with the live birth rate. Biochemical and clinical pregnancy rates increased from 36.14% to 55.77% (OR 0.499, 95% CI 0.221–0.910, *P* = 0.026; aOR 0.409, 95% CI 0.189–0.888, *P* = 0.024) and 33.73% to 50.00% (OR 0.509, 95% CI 0.251–1.034, *P* = 0.062; aOR 0.473, 95% CI 0.219–1.019, *P* = 0.056) comparing the eSET group to the DET group (Table 2).

Among the potential associated variables on the live birth rate, including age, BMI, AFC, EMT, the number of oocytes retrieved, and the number of transferred embryos, univariate and multivariate analyses showed that in POSEIDON Group 1, none of the above variables was found to be significantly associated with the live birth rate. However, for Group 2, age (OR 0.759, 95% CI 0.624-0.922, *P* = 0.006 and OR 0.751, 95% CI 0.605-0.932, *P* = 0.009) and the number of transferred (OR 0.412, 95% CI 0.190-0.892, *P* = 0.024 and OR 0.367, 95% CI 0.161-0.840, *P* = 0.018) embryos were shown to have a significant association with the live birth rate (Table 3).

**Table 3:** Logistic regression analysis on the live birth rate in patients with unexpected poor prognosis

	Group 1				Group 2			
	Univariate analysis		Multivariate analysis		Univariate analysis		Multivariate analysis	
	OR (95% CI)	*P* value	OR (95% CI)	*P* value	OR (95% CI)	*P* value	OR (95% CI)	*P* value
**Age**	0.959 (0.869–1.059)	0.409	0.951 (0.859-1.053)	0.336	0.759 (0.624–0.922)	0.006	0.751 (0.605–0.932)	0.009
**Body Mass Index / BMI**	0.969 (0.887–1.059)	0.490	0.973 (0.886-1.069)	0.573	0.900 (0.769–1.053)	0.188	0.939 (0.793–1.113)	0.471
**AFC**	0.986 (0.936–1.039)	0.598	0.983 (0.930-1.039)	0.547	1.042 (0.963–1.128)	0.302	1.017 (0.932–1.110)	0.699
**Endometrial thickness**	1.004 (0.894–1.127)	0.948	1.019 (0.905-1.147)	0.758	1.082 (0.907–1.291)	0.381	1.020 (0.836–1.246)	0.842
**No. oocytes retrieved**	1.076 (0.945–1.226)	0.266	1.059 (0.926-1.210)	0.404	1.007 (0.841–1.207)	0.939	0.979 (0.807–1.187)	0.827
**No. transferred embryos**	0.786 (0.462–1.337)	0.374	0.751 (0.427-1.321)	0.321	0.412 (0.190–0.892)	0.024	0.367 (0.161–0.840)	0.018

## Discussion

To the best of our knowledge, there was a lack of the discussion about the management of POSEIDON low-prognosis patients specific to the embryo transfer protocols, including the decisions on embryo quantity and quality. As it was described in the 2023 ESHRE guideline, there was no identified evidence for low responders with regards to eSET vs. DET [14]. Our study showed the novelty that the embryo number to transfer for the POSEIDON Group was discussed on for the first time. The current study analyzed the first FET with blastocyst transfer, and it was observed in POSEIDON Group 1 that compared to single blastocyst transfer, an upward trend was present among the clinical outcomes of double blastocyst transfer but without significance, which was accompanied by a significant increase of multiple birth rate. In POSEIDON Group 2, double blastocyst transfer was significantly associated with the improvement in clinical outcomes with multiple birth rate not significantly increased. Furthermore, age and the number of transferred embryos were demonstrated to be crucial variables for the live birth rate in unexpected low-prognosis patients ≥ 35 years.

POSEIDON unexpected low responders (i.e., Group 1&2) may raise our concerns about the potential negative effects of poor ovarian response on embryo transfer for two aspects. Firstly, POR with normal reserve is associated with the single-nucleotide polymorphism of FSHR [15]. And low responders with abnormal expression of FSHR were also demonstrated to be associated with attenuation of endogenous hormone synthesis in ovarian granulosa cells [16], which potentially affects follicle development, endometrium as well as implantation synchronism. Moreover, the correlation between ovarian response and follicle or embryo quality is debated. Morin et al. revealed that blastulation rate, aneuploidy rate and live birth rate were comparable between patients with oocyte yield in the < 10th percentile and those in the 25-75th percentile [17] However, Schachter-Safrai N et al. argued that embryos from patients with retrieved oocyte ≤ 5 reached the morphokinetic milestones later than embryos obtained from normal responders. In low responders, implanted embryos manifested a protracted course of blastocyst formation compared with implanted embryos from the normal responders [18]. Although there is not yet a consensus about this topic, there is a signal that poor ovarian response may impact embryo quality and endometrial receptivity, thus exploration of outcomes and strategies to improve outcomes in patients beyond response to ovarian stimulation is warranted.

Embryo transfer strategy, as the most crucial part of controlling multiple births, has been constantly discussed in different patient populations. For POSEIDON low responders, the availability of usable embryos is one of the prominent limitations for their clinical outcomes. Focusing on poor responders, an early study found double embryo transfer might lead to a higher live birth rate than those with single embryo transfer, with a modest increase in the multiple birth rate (9.1%) [19]. In 2015, Gleicher et al. retrospectively analyzed the live birth rates of transferring embryos in various numbers among poor responders by Bologna criteria. With age stratification, the results showed that young patients had a satisfactory live birth rate of 33% whether with non-elective one or two embryos transferred. Live birth rates in patients aged 35–37 and 38–40 seemed to associate with embryo number. For patients with advanced age, the rates were as low as 5.9%-7.4% even after three embryos were transferred [20].

Patient prognosis was considered one of the critical factors for the decisions on transfer protocols. Various studies investigated embryo transfer strategies for patients with different prognoses. However, the standard of prognosis was not consistently defined in these studies. Loendersloot et al. defined women ≤ 35 years who underwent their first IVF/ICSI cycle as good prognosis and women ≥ 39 years as poor prognosis. Women aged between 35–39 years, young patients undergoing their first IVF without a top-quality embryo, or having at least one failed IVF cycle were classified as intermediate prognosis [21]. Another study classified patients’ prognoses according to their previous IVF history and outcomes, as well as whether they had extra embryos for cryopreservation [22]. Gleicher et al. defined poor responders under Bologna criteria as a very poor prognosis population [20]. Since 2016, POSEIDON criteria have converted the conception of poor responder into low prognosis. Our study provided a novel comparative analysis under the POSEIDON low prognosis conception and the favorable clinical setting according to the 2021 ASRM/SART guideline.

In our study, POSEIDON Group 1 did not obviously benefit from double blastocysts transfer, which was associated with a slight increase of 6.02% in the live birth rate. However, the benefits were largely offset by the multiple birth rates significantly rising to 20.00% (Table 2, Fig. 2). It was worth mentioning that the live birth rate could reach 46.15% with elective single blastocyst transfer and 52.17% with double blastocyst transfer. Although defined as low prognosis by POSEIDON criteria, patients aged ≤ 35 years seemed to have quite acceptable outcomes. This information may provide some guidance for whether multiple ovum pick-up (OPU) cycles should be scheduled to accumulate embryos for improving patient outcomes. As reported, the blastulation rate of embryos and the live birth rates per embryo transfer cycle in young patients with low ovarian response were equivalent to those with a normal response, indicating that oocyte quality may not decline in this population [17]. It was demonstrated in a more recent study that euploidy rates in POSEIDON Group 1 were not significantly different from good-prognosis patients and were not influenced by the ovarian response [23]. POSEIDON Group 1, as the young unexpected low-prognosis patients, possibly could be regarded as good prognosis once they underwent blastocyst transfer in the first FET. Given that acceptable outcomes were observed in eSET and multiple birth rates raised in DET, this patient population should be encouraged to adopt single embryo transfer, as the 2021 ASRM/SART embryo transfer guideline recommended [7].

Our study demonstrated a significant association between embryo number and clinical outcomes in POSEIDON Group 2. Double blastocyst transfer showed its superiority in promoting the live birth rate from 20.48% to 38.46%. Logistic regression analysis suggested both age and embryo number as important variables in this group. Heterogeneity remained in the efficiency of different embryo transfer strategies for aged patients in the previous clinical research. A retrospective analysis including 155 patients with ages ≥ 35 years reported that the live birth rate of double blastocyst transfer did not differ from single blastocyst transfer (46.5% versus 37.0%, *P* = 0.228), whereas the risks of preterm birth and low birth weight increased [24]. Another retrospective cohort for patients ≥ 40 years demonstrated that compared to elective single blastocyst transfer, double blastocyst transfer showed a similar live birth rate (22.8% versus 20%, *P* = 0.20). However, elective double blastocyst transfer, which meant extra blastocyst available for cryopreservation, achieved significant improvement in the live birth rate (30.6% versus 20%, *P* = 0.017) [25]. The study targeting patients aged 40–44 years by Niinimäki, M et al. showed the opposite result that advanced patients had higher cumulative live birth rates with elective single embryo transfer than with double embryo transfer (22.7% versus 13.2%, *P* = 0.002) [26]. These studies did not further stratify the patients according to their prognosis, which was an important consideration for making a transfer strategy. For POSEIDON Group 2, it seemed that double embryo transfer would be preferred when merely discussing the live birth rate. Meanwhile, the potential accompanying risk of multiple births should be noticed.

With the restriction of local policies, cases of transferring more than two blastocysts were lacking. It is expected that data about clinical outcomes with multiple embryo transfers for POSEIDON patients could be discussed worldwide in the future. We also observed that many studies about embryo transfer refined the patients’ age when discussing the aged population, and Logistic regression demonstrated the significance of age for the live birth rate. It may inspire us to further stratify the patients’ age in the following study to test the results in each refining age group. Our study was also limited in the nature of the retrospective design with inherent bias and unknown confounders. To avoid the bias from diseases with significant impact on clinical outcomes, including recurrent miscarriage, chromosomal abnormality, and hereditary diseases, patients with PGD/PGS were eliminated, which may refer to patients with older age and potentially influence the composition of patient age. However, there was no significant difference in age between eSET and DET within two groups, weakening the impact on the comparability. The relatively smaller sample size of Group 2 could be explained by, 1) The proportion of poor-quality or impotent embryos was higher in older patients, limiting the blastulation rate and the acessibility of usable blastocysts. 2) Ovarian reserve is diminishing by age naturally. Older patients with normal ovarian reserve parameters (AMH≥1.2ng/ml and/or AFC ≥5) would be less common than the younger. And this limitation may weaken the analysis’s statistical power and the conclusion should be deducted and interpreted with caution. Another limitation was that only POSEIDON Group 1 and Group 2 were included in the present study given that clinical data of POSEIDON Group 3 and Group 4 were relatively fewer for analysis and collecting work was still ongoing. A large-scale multi-center prospective analysis is expected to further investigate the embryo transfer strategy for POSEIDON patients.

In conclusion, following the embryo transfer guideline, we advocated single blastocyst transfer as the first option in the first FET cycle for POSEIDON Group 1. POSEIDON Group 2 could benefit from double embryo transfer. When multiple embryo transfer was selected, the risk of multiple birth rates should be considered and personal willingness, basic health conditions as well as economic factors should be fully evaluated. A more scientific discussion about the embryo transfer strategy under POSEIDON criteria might base on a reasonable age stratification. More precise management based on POSEIDON criteria may be the treatment strategy for poor responders in the future.

### Supplementary Information

Supplementary Material 1: Supplementary Table 1. Baseline clinical characteristics of patients with unexpected poor prognosis.

## Data Availability

The data underlying this article will be shared on reasonable request to the corresponding author.

## Abbreviations

eSET: Elective single embryo transfer
DET: Double embryo transfer
ART: Assisted reproductive technology
DOR: Diminished ovarian reserve
POR: Poor ovarian response
AFC: Antral follicle count;
AMH: Anti-mullerian hormone
IVF: In vitro fertilization
FET: Frozen embryo transfer
PGS/PGD: Preimplantation genetic screening/diagnosis
ICM: Inner cell mass
TE: Trophectoderm
BMI: Body mass index
EMT: Endometrium thickness
Gn: Gonadotropin
COH: Controlled ovarian hyperstimulation
