# Supplementary material for: What is the optimal number of embryos to transfer for POSEIDON group 1 and group 2? A retrospective study
Source: J Ovarian Res. 2024 May 31;17:117. doi: 10.1186/s13048-024-01443-y (PMC11140960; doi:10.1186/s13048-024-01443-y)
Supplement: Supplementary file 1 — Supplementary Material 1: Supplementary Table 1. Baseline clinical characteristics of patients with unexpected poor prognosis. [file 13048_2024_1443_MOESM1_ESM.docx]

**Supplementary Table 1. Baseline clinical characteristics of patients with unexpected poor prognosis.**

|  | **Group 1 (n=219)** | **Group 2 (n=135)** | ***P* value** |
| --- | --- | --- | --- |
| **Age (years)** | 30.68±2.70 | 37.87±2.52 | 0.000 |
| **BMI (kg/m2)** | 21.18±3.02 | 22.14±2.53 | 0.002 |
| **Duration of infertility(years)** | 3.37±1.99 | 3.93±3.17 | 0.063 |
| **Indications for IVF** |  | | |
| tubal factor | 39.27% | 46.67% | 0.028 |
| endometriosis | 8.68% | 1.48% |  |
| male factor | 10.50% | 15.56% |  |
| combined factors | 7.31% | 5.19% |  |
| unknown factors | 34.25% | 31.11% |  |
| **AFC** | 12.01±5.13 | 9.95±4.65 | 0.000 |
| **FSH (IU/L)** | 7.84±2.18 | 8.07±2.45 | 0.361 |
| **LH (IU/L)** | 4.88±2.78 | 4.21±1.85 | 0.007 |
| **E2 (pg/mL)** | 49.30±45.55 | 48.60±28.64 | 0.873 |
| **P (ng/mL)** | 0.76±1.23 | 0.76±1.28 | 0.981 |
| **PRL (ng/mL)** | 21.24±42.03 | 21.04±32.31 | 0.964 |
| **T (ng/mL)** | 0.98±5.05 | 0.83±3.03 | 0.757 |
| **AMH (ng/mL)** | 3.77±2.25 | 3.00±2.32 | 0.002 |
| **COH protocol** |  | | |
| GnRH-Agonist | 27.85% | 28.89% | 0.834 |
| GnRH-Antagonist | 72.15% | 71.11% |  |
| **Total Gn dose (IU)** | 2454.37±1028.87 | 2980.56±1161.65 | 0.000 |
| **No. Oocytes retrieved** | 6.66±2.07 | 6.44±2.10 | 0.333 |

BMI, Body Mass Index; IVF, in vitro fertilization; AFC, antral follicle count; FSH, Follicle-Stimulating Hormone; LH, Luteinizing Hormone; E2, Estradiol; P, Progesterone; PRL, prolactin; T, testosterone; AMH, anti-mullerian hormone, COH, controlled ovarian hyperstimulation; Gn, Gonadotropin
